# Neurodevelopmental Outcomes in Preterm Babies: A 12-Month Observational Study

**DOI:** 10.7759/cureus.47775

**Published:** 2023-10-27

**Authors:** Shresth Jain, Putun Patel, Nimisha Pandya, Dhruva Dave, Trupti Deshpande

**Affiliations:** 1 Pediatric Medicine, Gujarat Medical Education and Research Society (GMERS) Medical College and Hospital, Gotri, Vadodara, IND; 2 Pediatric Intensive Care Unit, Gujarat Medical Education and Research Society (GMERS) Medical College and Hospital, Gotri, Vadodara, IND

**Keywords:** observational study, neonatal intensive care unit (nicu), preterm babies, neurodevelopmental outcome, extremely low birth weight infant

## Abstract

Background

Preterm births are a significant concern worldwide due to their association with both short- and long-term morbidity. Modern neonatal intensive care techniques have improved the survival of infants born at the brink of viability. However, there remain significant challenges concerning their neurodevelopment. A considerable proportion of very low birth weight infants exhibit significant motor deficits such as cerebral palsy or cognitive, behavioral, or attention disabilities. The consequences of these impairments, particularly given their life-long nature, can be severe for the affected individuals, families, and public health resources. Consequently, timely neurodevelopmental assessment is critical in recognizing delayed development and selecting infants for neurodevelopmental stimulation. This study aimed to estimate the neurodevelopment of preterm infants, identify influencing factors, detect at-risk groups, and refer/recommend early intervention when developmental delays are observed.

Methodology

This prospective, observational, hospital-based study done in the department of pediatrics, Gujarat Medical Education and Research Society (GMERS) Medical College and Hospital, Gotri, Vadodara, Gujrat, India included inborn and outborn preterm neonates admitted to the Neonatal Intensive Care Unit (NICU) or the Sick Newborn Care Unit from their first day of life. The study period was from October 2020 to January 2021, and only neonates with an uncomplicated clinical course were included. Newborns were enrolled in a high-risk clinic, and follow-up appointments were scheduled at three, six, nine, and 12 months of corrected gestational age (CGA). We used the Baroda Developmental Screening Tool (BDST) to calculate the developmental quotient (DQ) at each appointment. This assessment involved parental interviews, observation of developmental milestones, and simple test demonstrations. The gathered DQ data at different ages were analyzed and compared across groups.

Results

Of 100 preterms enrolled, 62 preterms were followed up until 12 months of CGA. Thirteen patients out of the 62 (approximately one-fifth) preterm neonates exhibited developmental delays at one year of CGA, most of whom were early preterm infants. Twenty-six patients (approximately two-fifths) were delayed at three months of CGA, and thus 13 patients (half) showed catch-up growth and development. There was no statistically significant difference between the neurodevelopment of female and male infants. However, infants born to mothers with better socioeconomic status and higher education showed improved neurodevelopment.

Conclusions

Our study findings suggest that preterm infants discharged from the NICU exhibit poor neurodevelopmental outcomes, especially those born early preterm. This pattern indicates an inverse relationship between neurodevelopmental delay and the maturity of the neonate. Maternal education and socioeconomic status positively impacted the neurodevelopment of preterm NICU graduates. Thus, regular follow-up (at least once every three months), early detection by a screening scale like the BDST and intervention significantly improved neurodevelopmental outcomes.

## Introduction

Preterm births, defined as those occurring before the completion of 37 weeks of gestation according to the World Health Organization, significantly contribute to both short- and long-term morbidity [1-3]. These births are further classified as extremely preterm (less than 28 weeks), early preterm (28 to less than 32 weeks), and moderate to late preterm (32 to less than 37 weeks) [1]. Of the 27 million registered newborns delivered annually in India, the incidence of premature birth was 3.5 million [1], with 85% occurring between 32 to 37 weeks of gestation [4]. Most extremely low birth weight infants are born to women of low socioeconomic status, teenagers, or women older than 40 [5,6].

The primary causes of preterm birth include multiple pregnancies, maternal infections, chronic conditions like diabetes and hypertension, and genetic factors [7]. Crucial clinical outcomes for preterm infants encompass survival and normal long-term neurodevelopment for independent survival in the future. However, numerous preterm survivors encounter a range of disabilities, including cerebral palsy, sensory deficits, and learning disabilities [8,9]. Preterm children are at an elevated risk for language, cognitive, sensory, and motor impairments, which can result in poor academic performance or behavioral issues at school and adversely affect the socioeconomic life of individuals and families [10,11].

These debilitating effects can severely impact individuals and their families. Consequently, current neonatal interventions prioritize “intact survival,” defined as survival without disabilities [12]. Neurodevelopmental assessment is vital as it timely identifies delayed development, facilitating early neurodevelopmental stimulation and improved outcomes.

The domains of development include gross motor development, fine motor development, social/cognitive/intellectual development, speech and language development, and vision and hearing development. In India, several scales like the Baroda Developmental Screening Tool (BDST) [13], Trivandrum Developmental Screening Chart [14], and the Lucknow Development Screen for Indian Infants [15] are employed to assess these domains.

This study estimated the neurodevelopment of preterm infants discharged from the Neonatal Intensive Care Unit (NICU) at three, six, nine, and 12 months of corrected gestational age (CGA). Further, the study intended to compare the development of preterm infants appropriate for gestational age (AGA) with preterm small for gestational age (SGA) infants, assess differences between preterm girls and boys, and contrast early and late preterm development. The research also sought to identify factors impacting neurodevelopment. This study helped to reflect the effect of socioeconomic status of mothers with preterm neurodevelopmental outcomes and initiate early intervention in cases of delayed development.

## Materials and methods

The present study was conducted as a prospective observational study at the NICU at the Department of Paediatrics, Gujarat Medical Education and Research Society Medical College, Gotri, Vadodara, Gujrat, India. The study was carried out from October 2020 to January 2022 after obtaining approval from the scientific and ethics committee (Approval No. GMERS Medi 551/09/2020). The study was conducted on 100 preterm infants with a relatively uncomplicated stay after obtaining consent from their parents or caregivers. Those babies with hypoxic-ischaemic encephalopathy (HIE)/intraventricular hemorrhage (IVH), meningitis, babies on prolonged ventilation or continuous positive airway pressure (CPAP) for more than seven days, babies with obvious syndromic features, and babies requiring surgery were excluded from the study. 

The sample size was calculated assuming the expected population standard deviation to be 10, and employing t-distribution to estimate sample size, the study would require a sample size of 28 to estimate a mean with 95% confidence and a precision of 4. Considering attrition of up to 40% on follow-up till 18 months, the sample size will be 46. The 28 is 60% of 46. therefore, early preterm is 46, late preterm is 46, leading to a total of 92, rounded off approximately to 100 babies. The calculation was done using an online sample size calculator.

All preterm newborns less than 37 weeks of gestational age satisfying inclusion criteria admitted in NICU, GMERS Hospital, Vadodara between October 2020 and January 2021 were registered along with the contact number of the parents/caregiver was obtained at admission and were reminded for follow-up after discharge by telephonic method. Gestational age was assessed using a modified Ballard score and was done in the first 24 hours of birth. Preterm infants enrolled were divided into two groups: less than 32 weeks (early preterm) and more than 32 weeks (late preterm). Each of these classes was divided into SGA, AGA, and LGA respectively using Fenton charts. 

Maternal history viz. educational status, social status (modified Kuppuswamy scale), nutritional status (BMI), the status of work, and any significant history was obtained. Newborns admitted and discharged from NICU were called for follow-up at three, six, nine, and 12 months of corrected age (adjusted for prematurity) in HRC which is on Friday mornings 9-12 am. Detailed history including history of milestones, anthropometry, general examination, and neurological examination were recorded on follow-up. BDST was performed by asking questions to parents, observing milestones, and demonstrating simple tests at each follow-up. We employed the BDST due to its validation for Indian infants, minimal training requirements, short administration time, and impressive psychometric properties [16,17]. The Developmental Quotient (DQ) was calculated using the BDST [13], and various groups’ DQs were compared. We excluded the parental questionnaire because of significant discrepancies between parental milestones reports and actual milestones demonstrated by infants.

All the variables will be grouped as per mathematic transformation into nominal/ordinal/interval and ratio. Further point estimates with dispersion measures will be calculated with the help of Excel® (Microsoft, Redmond, WA, USA) and other statistical software. The extent of type one error will be measured with parametric analysis. Z test will be applied for proportion and the t-test (unpaired) will be used to find out any significance difference between detected proportion and mean This univariate analysis will be further treated with multivariate analysis logistic/linear regression at appropriate places.

## Results

Sample distribution and comparison

This study incorporated 100 neonates, evenly divided between early and late preterm. From this population, 62 patients continued with follow-up until one year of CGA, including 33 late preterm and 29 early preterm infants. Out of 38 patients, six died, resulting in 32 patients lost to follow-up.

The average birth weight of the patients in this study was 1.49 ± 0.35 kg. Their mean developmental age was 11.51 ± 2.22 months. The mean DQ of patients at three, six, nine, and 12 months was 92.28 ± 30.01%, 95.46 ± 26.74%, 94.26 ± 21.01%, and 95.89 ± 18.47%, respectively.

The study’s population comprised 62 patients of which 42 were AGA and 20 SGA. The difference between the DQ means for AGA and SGA was statistically significant at three and six months (p<0.05) but not significant at nine and 12 months (Table 1). The odds ratio (OR; 0.9166) with 95% confidence interval (CI), did not indicate either group as a risk factor.

**Table 1:** Comparison of Developmental Quotient (DQ) of AGA and SGA Abbreviations: AGA, appropriate for gestational age; DA, developmentally adequate; DD, developmentally delayed; OR, odds ratio; SGA, short for gestational age.

Age	Newborns Nutritional Status	Mean	P-value	DD (n)	DA (n)	OR (CI = 95%)	Significance level
3 months	AGA	87.14	0.0497	20	22	0.9166	P = 0.8972
	SGA	103.08		6	14		
6 months	AGA	90.71	0.0419	20	22		
	SGA	105.42		6	14		
9 months	AGA	92.21	0.2695	8	34		
	SGA	98.56		4	16		
12 months	AGA	94.24	0.3095	9	33		
	SGA	99.38		4	16		

The sex distribution included 34 female infants and 28 male infants. Although the mean DQ of male infants was higher than that of females (Table 2), the difference was not statistically significant. The OR of 14.727 with 95% CI suggests that female infants had a greater risk of delayed development in this study than male infants.

**Table 2:** Comparison of Developmental Quotient (DQ) according to sex Abbreviations: DA, developmentally adequate; DD, developmentally delayed; OR, odds ratio.

Age	Sex	Mean	P-value	DD (n)	DA (n)	OR (CI = 95%)	Significance level
3 months	Female	86.62	0.1019	17	17	14.727	0.0127
	Male	99.16		9	19		
6 months	Female	92.65	0.3664	16	18		
	Male	98.87		10	18		
9 months	Female	91.07	0.1909	10	24		
	Male	98.12		2	26		
12 months	Female	92.28	0.0898	12	22		
	Male	100.28		1	27		

Of the 62 follow-up patients, 29 were early preterm, and 33 were late preterm. The mean DQ of late preterm infants was higher than that of early preterm infants (Table 3), a statistically significant difference (p<.001). An OR of 22.5882 with 95% CI implies that early preterm infants had a higher risk for neurodevelopmental delay than late preterm infants.

**Table 3:** Comparison of Developmental Quotient (DQ) of early and late preterm Abbreviations: DA, developmentally adequate; DD, developmentally delayed; OR, odds ratio.

Age	Gestational Age Stage	Mean	P-value	DD	DA	OR (CI = 95 %)	Significance level
3 months	Early	68.16	< .001>	23	6	22.5882	P= 0.0040
	Late	113.49		3	30		
6 months	Early	73.62	< .001>	23	6		
	Late	114.65		3	30		
9 months	Early	78.33	< .001>	11	18		
	Late	108.25		1	32		
12 months	Early	81.88	< .001>	12	17		
	Late	108.21		1	32		

Concerning socioeconomic status, three, 32, 25, and two patients belonged to the lower, upper-lower, lower-middle, and upper-middle class, respectively. Application of the ANOVA test revealed no statistically significant difference between the means of all groups (Table 4). Similarly, the t-test applied to means of two groups formed by various combinations also demonstrated no statistically significant difference (Table 5).

**Table 4:** Comparison of Developmental Quotient (DQ) according to maternal social status

Age	Maternal Social Status	Frequency	Mean	f-value
12 months	Lower Class	3	84.72	0.414
	Upper Lower Class	32	100.15	
	Lower Middle Class	25	91.67	
	Upper Middle Class	2	93.88	

**Table 5:** P-values between means of Developmental Quotient (DQ) of various socioeconomic status (SES) groups

T-test between mean DQ of SES Groups	3 Months	6 Months	9 Months	12 Months
Lower Class & Upper Lower	P = 0.2293	P = 0.4984	P = 0.5742	P = 0.5156
Lower Class & Lower Middle Class	P = 0.1979	P = 0.1728	P = 0.2625	P = 0.1940
Lower Class & Upper Middle Class	P = 0.6742	P = 0.5204	P = 0.6295	P = 0.6477
Upper Lower & Lower Middle Class	P = 0.2411	P = 0.2561	P = 0.2134	P = 0.2864
Upper Lower & Upper Middle Class	P = 0.7136	P = 0.9873	P = 0.8604	P = 0.8978
Lower Middle Class & Upper Middle Class	P = 0.5342	P = 0.6321	P = 0.7759	P = 0.5548

In the sample population, 17 mothers had not completed primary education, 32 had achieved primary education, 12 had secondary education, and one graduate mother. The ANOVA test revealed no significant difference between the means of all groups (Table 6). However, the t-test between means of two groups formed by different combinations showed a statistically significant difference between means of DQ of infants born to uneducated and primarily educated mothers, as well as uneducated and secondarily educated mothers, at three, six, nine, and 12 months of corrected age (Table 7).

**Table 6:** Comparison of Developmental Quotient (DQ) according to maternal educational status

Age	Educational Status	Frequency (n)	Mean DQ	f-value
12 months	Uneducated	17	85.29	0.0951
	Primary	32	99.47	
	Secondary	12	103.12	
	Graduate	1	75.00	

**Table 7:** P-values between mean Developmental Quotient (DQ) of maternal educational levels. Abbreviations: NA, not applicable (i.e., the mean DQ of babies of graduate mother can be neglected as only one mother was graduate; sample size is too small to comment).

T-test between Mean DQ of Educational Levels	3 Months	6 Months	9 Months	12 Months
Uneducated & Primary	P = 0.0224	P = 0.0249	P = 0.0122	P = 0.0086
Uneducated & Secondary	P = 0.2552	P = 0.0210	P = 0.0168	P = 0.0112
Uneducated & Graduate	N.A.	N.A.	N.A.	N.A.
Primary & Secondary	P = 0.4061	P = 0.6569	P = 0.5875	P = 0.5396
Primary & Graduate	N.A.	N.A.	N.A.	N.A.
Secondary & Graduate	N.A.	N.A.	N.A.	N.A.

Of all the mothers, 43 were homemakers, while 19 had employment outside the home. The mean DQ of infants born to homemakers was higher than that of working mothers (Table 8). However, no statistically significant difference was found between these means.

**Table 8:** Comparison of Developmental Quotient (DQ) of babies of mother by employment status

Months	Status of Working	Mean	P-value
12 months	Homemaker	96.79	0.5689
	Employed outside the home	93.86	

At three months of CGA, 36 patients exhibited adequate development, while 26 showed developmental delay. Among the delayed patients, three were late preterm, and 23 were early preterm. Upon assessment at 12 months of CGA, 49 patients exhibited adequate development, and 13 were delayed. Of these delayed patients, only one was late preterm, and 12 were early preterm (Table 9).

**Table 9:** Developmental outcome Abbreviations: DA, developmentally adequate; DD, developmentally delayed.

Population Category	Result	3 Months (n)	6 Months (n)	9 Months (n)	12 Months (n)
Total Population	DA	36	36	50	49
	DD	26	26	12	13
Early	DA	6	6	18	17
	DD	23	23	11	12
Late	DA	30	30	32	32
	DD	3	3	1	1

## Discussion

Preterm birth significantly contributes to morbidity and mortality in children under five globally, particularly in South Asia and Africa, which bear the brunt of developmental disabilities [10,11]. Our research contributes to the existing literature that underscores the prevalence of developmental delays in preterm infants [18,19].

The early detection of developmental disabilities can pave the way for prompt interventions. Such interventions potentially enhance the DQ and prognosis of patients. 

The study results found that late preterms showed the highest DQ, whereas early preterms demonstrated the lowest at one year. These findings are attributable to effective practices in the NICU, such as early feeding, Kangaroo Mother Care, surfactant application, mechanical ventilation, regular high-risk clinic follow-ups, and exclusive breastfeeding (EBF) [20].

Interestingly, our study reported more AGA than SGA neonates, all with a normal mean DQ at one year of CGA. We noted a significant difference between the DQs of AGA and SGA neonates at three and six months, with SGA neonates performing better. However, this difference became insignificant at nine and 12 months, suggesting AGA infants experienced catch-up growth.

Our findings contrast with those of Rani et al. [21] and Murray et al. [22], which reported poorer performance among SGA infants. This discrepancy might stem from varied care quality across different Indian settings, which impacts neurodevelopment outcomes. While AGA neonates often receive surfactant therapy and oxygen support, SGA infants are more susceptible to metabolic disorders and necrotizing enterocolitis whose prognosis is poor in the Indian setting [23].

Contrary to global studies, we found female infants at a higher risk of delayed development than male infants [24,25]. This disparity could be due to societal gender bias [26] or the fact that most developmentally delayed female infants in our study were early preterm. Their compromised neurodevelopment could thus be primarily due to their premature birth status.

We also observed that parents more frequently brought male children for follow-up visits, possibly reflecting the prevalent gender bias in Indian society. As India is a low-income country, where many lack robust socioeconomic status (SES) and literacy, some families may hold biases favoring male children, which could result in fewer follow-up visits for female children.

Regarding socioeconomic status, infants born to mothers from the lower class exhibited DQs below the cut-off at three months of CGA. However, these scores normalized by the end of the first year, suggesting a catch-up growth. Lower socioeconomic status might lead to poorer outcomes due to differences in child-rearing practices, resource scarcity, and reduced maternal attention.

We found a significant difference in the DQs of babies born to uneducated mothers and those with primary or secondary education. This variation may result from increased awareness among more educated mothers about practices like breastfeeding, hypothermia prevention, sanitation, and immunization. Despite the normal mean DQs for infants born to both working mothers and homemakers, our results may have been influenced by the mothers’ educational levels. Notably, our study aligns with findings from González et al. [27] and Patra et al. [28], showing the significant influence of social class and mother’s educational level on neonatal neurodevelopment.

In our study, 20.97% of preterm infants displayed developmental delays according to the BDST criteria [13]. Of the 62 preterms, 26 showed a DQ below the cut-off at three and six months of age, but only 13 remained below the cut-off at one year. This reduction underscores the effectiveness of early interventions like stimulation, and physiotherapy and demonstrates the role of neuroplasticity.

However, our study has some limitations as it was a single center. We did not assess the long-term impact of prematurity on neurodevelopment or potential psychiatric or psychological disorders including behavioral disorders. This omission restricts our ability to extrapolate the long-term outcomes based on the current findings. Moreover, our sample was limited to neurologically stable neonates, excluding infants with more severe health conditions such as central nervous system infections or those requiring prolonged ventilation. This exclusion criterion likely narrows the generalizability of our findings to all neonates discharged from the NICU. Despite the relatively small sample size, we could definitively conclude that early preterm birth leads to a poor prognosis and that both socioeconomic status and maternal education positively influence outcomes. However, we could not definitively ascertain whether male or female infants have a worse or better prognosis. A larger sample size, enabling matching or randomization of male and female infants in both early and late preterm categories, would enhance the robustness of our insights into gender-based outcomes. Moreover, we did not differentiate the type of employment among mothers categorized as employed, instead only distinguishing between homemakers and those employed outside the home. Though we initially presumed that the type of employment would be indirectly covered by socioeconomic status or educational level, this assumption does not always hold true. Employment type may affect the amount of quality time a mother can spend with her child, potentially impacting the child’s neurodevelopment. Additionally, a child with a well-employed mother could exhibit poor neurodevelopment due to being early preterm. However, our follow-up might have included that same child because the parents were more health-conscious. To disentangle these confounding factors, a larger sample size and careful matching are required to attribute causes to observed outcomes accurately. These limitations should be considered when interpreting our results and their applicability to broader populations.

## Conclusions

Our study’s results indicate that infants born prematurely and discharged from the NICU tend to display compromised neurodevelopmental outcomes. This pattern is particularly evident among early preterm infants, signifying an inverse relationship between neurodevelopmental delay and the neonate’s gestational age at birth. Furthermore, regular follow-up assessments, help in the early detection of developmental delays, and timely intervention in improving these outcomes.

The findings support the importance of policy measures for adolescent females who represent future mothers. Such policies should emphasize improving nutritional status, educational attainment, and overall socioeconomic standing as potential avenues for enhancing the neurodevelopmental outcomes of preterm infants. The necessity of timely follow-up, screening processes for early identification of developmental delays, and immediate intervention helps in good neurodevelopmental outcomes.

This underscores the importance of policy measures integrating comprehensive infrastructure, high-quality NICU practices, maternal health and nutritional programs, lactation support, and promoting Kangaroo Mother Care. Moreover, providing effective counseling services and underscoring the importance of EBF and regular follow-up assessments in HRCs can play pivotal roles in optimizing neurodevelopmental outcomes for preterm infants. Our findings suggest that such a multifaceted approach can enhance the developmental trajectory of preterm NICU graduates and may mitigate some of the adverse effects associated with premature birth.

A detailed study between each group (eg. female and male, AGA and SGA) with a large sample size and matching to decrease confounding factors is recommended for future studies.
